# Biliary sepsis complication with congenital hepatic fibrosis: an unexpected outcome

**DOI:** 10.1186/s12879-023-08681-3

**Published:** 2023-10-23

**Authors:** Jiawei Sun, Xiaoxiao Mi, Xiaoying Ye, Yiling ShenTu, Chun Liu, Dong Tang, WenJun Yang, Jie Yang, Xiaoping Ye, Xiaojie Ma, Junping Shi, Gongying Chen, Ling Gong

**Affiliations:** 1 Hangzhou Normal University, Zhejiang, China; 2 Institute of Translational Medicine, The Affiliated Hospital of Hangzhou Normal University, Zhejiang, China; 3 Department of Respiratory Medicine, Fuyang First People’s Hospital, Hangzhou, China; 4 Department of Medical Imaging (Radiology), The Affiliated Hospital of Hangzhou Normal University, Zhejiang, China; 5 Department of Pathology, The Affiliated Hospital of Hangzhou Normal University, Zhejiang, China; 6 Department of Infectious Disease (Liver Diseases), Lishui Municipal Central Hospital, Zhejiang, China; 7 Department of Infectious Disease (Liver Diseases), The Affiliated Hospital of Hangzhou Normal University, Zhejiang, 310015 China

**Keywords:** Congenital hepatic fibrosis, Biliary sepsis, Klebsiella pneumoniae, PKHD1

## Abstract

**Background:**

CHF (Congenital hepatic fibrosis) is a rare hereditary disease characterized by periportal fibrosis and ductal plate malformation. Little is known about the clinical presentations and outcome in CHF patients with an extraordinary complication with biliary sepsis.

**Case summary:**

Our case described a 23-year-old female diagnosed as CHF combined with biliary sepsis. Her blood culture was positive for KP (Klebsiella pneumoniae), and with a high level of CA19-9 (> 1200.00 U/ml, ref: <37.00 U/ml). Meanwhile, her imaging examinations showed intrahepatic bile duct dilatation, portal hypertension, splenomegaly, and renal cysts. Liver pathology revealed periportal fibrosis and irregularly shaped proliferating bile ducts. Whole-exome sequencing identified two heterozygous missense variants c.3860T > G (p. V1287G) and c.9059T > C (p. L3020P) in PKHD1 gene. After biliary sepsis relieved, her liver function test was normal, and imaging examination results showed no significant difference with the results harvested during her biliary sepsis occurred.

**Conclusion:**

The diagnosis of CHF complicated with biliary sepsis in the patient was made. Severely biliary sepsis due to KP infection may not inevitably aggravate congential liver abnormality in young patients. Our case provides a good reference for timely treatment of CHF patients with biliary sepsis.

## Introduction

CHF (Congenital hepatic fibrosis) is a rare hereditary fibropolycystic disease, both autosomal dominant and recessive inheritance patterns are common. The term “congenital hepatic fibrosis” was first introduced by Kerr et al. in 1961 [1]. The incidence is from 1 to 10,000 to 20,000 [2], with an increased risk among the offspring of such consanguineous marriages. Isolated CHF is rare, and it is usually associated with other syndromes such as Caroli disease, autosomal recessive polycystic kidney disease, autosomal dominant polycystic kidney disease, Meckel-Gruber syndrome, Joubert syndrome, and Bardet-Biedl syndrome [3]. The clinical presentation of CHF is widely varied, ranging from asymptomatic phase to life-threating conditions related to portal hypertension and hepatic decompensation. The disease is generally classified into four forms based on clinical features: portal hypertension, which is more frequently seen, cholangitis, mixed, and latent [4]. CHF is characterized by the ductal plate malformation (DPM) of interlobular bile ducts, and DPM causes the bile ducts to become dilated or cystic, impairing their ability to maintain normal bile flow through the entire biliary tract, resulting in biliary stasis [5]. Therefore, CHF patients could present with cholangitis, especially with severely sepsis, a rare and life-threatening complication, when infected with microorganism.

We present here a case of CHF complicating biliary sepsis. The patient was diagnosed as having biliary sepsis due to a high level of CA19-9 and blood culture positive for KP (Klebsiella pneumoniae). And the diagnosis of CHF was made according to the results from her liver biopsy, imaging examinations as well as whole-exome sequencing. Since CHF complicated with severely biliary sepsis have been rarely reported, here, we summarize the clinical characteristics of the patient, as well as follow-up outcome of the patient.

## Case presentation

On July 8, 2022, a 23-year-old female patient presented with fever and diarrhea on admission. Physical examination revealed enlarged spleen 3 cm below the left costal margin. She denied any previous history of diseases. Her family history showed no consanguineous marriages.

Blood routine examination showed leukocytosis (WBC, white blood cell 18 × 10^9/L, ref: 3.50–9.50 × 10^9/L), thrombocytopenia (PLT, platelet, 39 × 10^9/L, ref:125–350 × 10^9/L), increased levels of ultrasensitive CRP (C-reactive protein, 288.5 mg/L, ref:<10 mg/L) and PCT (Pre-calcitoninogen, 42.6 ng/ml, ref: <0.5 ng/ml). Biochemical tests showed elevated levels of ALT ( alanine transaminase, 59 U/L, ref: 0–40 U/L), AST ( aspartate aminotransferase, 72 U/L, ref: 0–40 U/L), TBIL ( total bilirubin, 26 µmol/L, ref: 0–23 µmol/L), DBIL (direct bilirubin, 11.7µmol/L, ref: 0–4 µmol/L), TBA ( total bile acid, 119.1 µmol/L, ref: 0–10 µmol/L), SCr ( creatinine, 241 µmol/L, ref: 21–75 µmol/L), and decreased level of ALB ( albumin, 29 g/L, ref: 40–55 g/L). Serum liver fibrosis indicators showed elevated levels of HA ( hyaluronic acid, 502 ng/ml, ref: <100 ng/mL), PCIII ( Type III collagen, 33.2 ng/ml, ref: <30 ng/ml), and IV-C (Collagen type IV, 41. 2 ng/ml, ref: <30 ng/ml). Serum tumor factors tests showed high levels of CA19-9 (Carbohydrate antigen 199,>1200 U/ml, ref: <39 U/ml) and CA125 (Carbohydrate antigen 125, 80.8 U/ml, ref: <39 U/ml). Serologic tests for viral hepatitis, autoantibodies, and platelet antibodies were negative. Serum IgM (Immunoglobulin M) and IgG (Immunoglobulin G) levels were normal. Serum ceruloplasmin levels and 24-hour urinary copper levels were normal, and ophthalmological examination revealed no evidence of the KF ring ( Kayser-Fleischer Ring ). Serum-ferritin, serum-iron and serum-total iron binding capacity were also all within normal ranges. Common liver diseases mainly include viral hepatitis, autoimmune hepatitis, drug-induced liver damage, metabolic liver disease including Wilson’s disease and hemochromatosis were excluded. Blood culture was positive for KP, and stool culture was positive for Candida albicans.

**Fig. 1:**
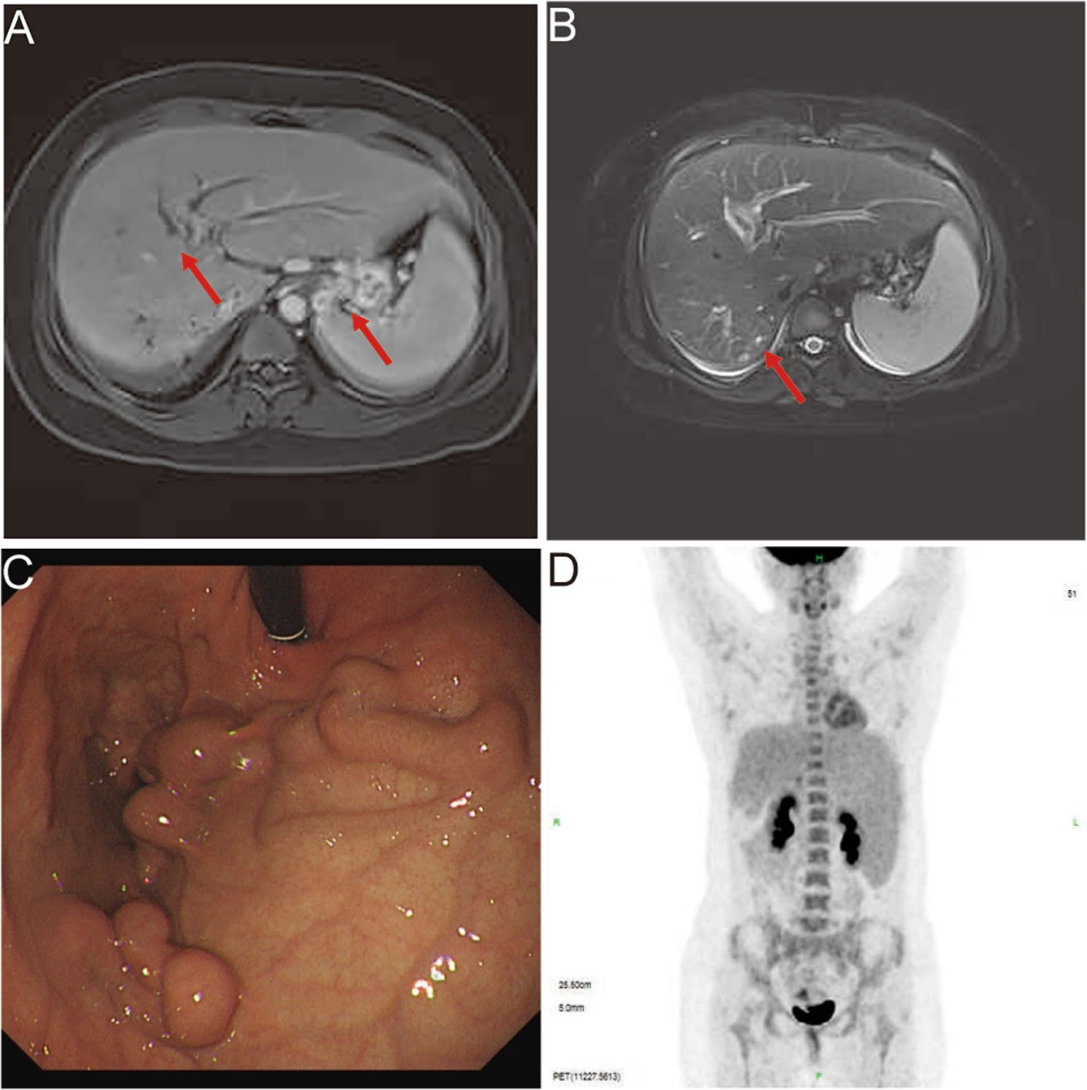
Imaging findings of the patient. **(A-B)** MRI imaging revealed dilated intrahepatic bile duct dilatation, gastric fundic varices, an enlarged spleen and small cyst in the kidney (arrow). **(C)** Upper gastrointestinal endoscopy revealed gastric fundic varices. **(D)** PET/CT imaging revealed slightly enlarged liver, hyperplastic lymph nodes around the portal vein, giant spleen with no increase in FDG metabolism and multiple varices in the fundic and retroperitoneum

Further, CT (Computed tomography) imaging revealed slight striate shadow in the lower lobes of both lungs, a slight enlargement of the liver’s left lobe, mild dilation of the intrahepatic bile ducts, splenomegaly, esophageal and gastric fundus varices, and numerous tiny cysts in both kidneys. MRI (magnetic resonance imaging) revealed dilated intrahepatic and common bile ducts (Fig. 1 A-B). Upper gastrointestinal endoscopy revealed gastric fundus varices without red signs (Fig. 1 C). Since the patient had elevated serum tumor markers (CA19-9 level>1200 U/ml, ref: <39 U/ml) and CA125 (80.8 U/ml, ref: <39 U/ml), with abnormal levels of ALT and AST, so PET/CT (positron emission tomography/computed tomography) was performed to exclude tumors. PET/CT findings suggested slightly enlarged liver with mild dilatation of the intrahepatic bile ducts, hyperplastic lymph nodes around the portal vein, giant spleen with no increase in FDG (fluorodeoxyglucose) metabolism (Fig. 1 D).

**Fig. 2:**
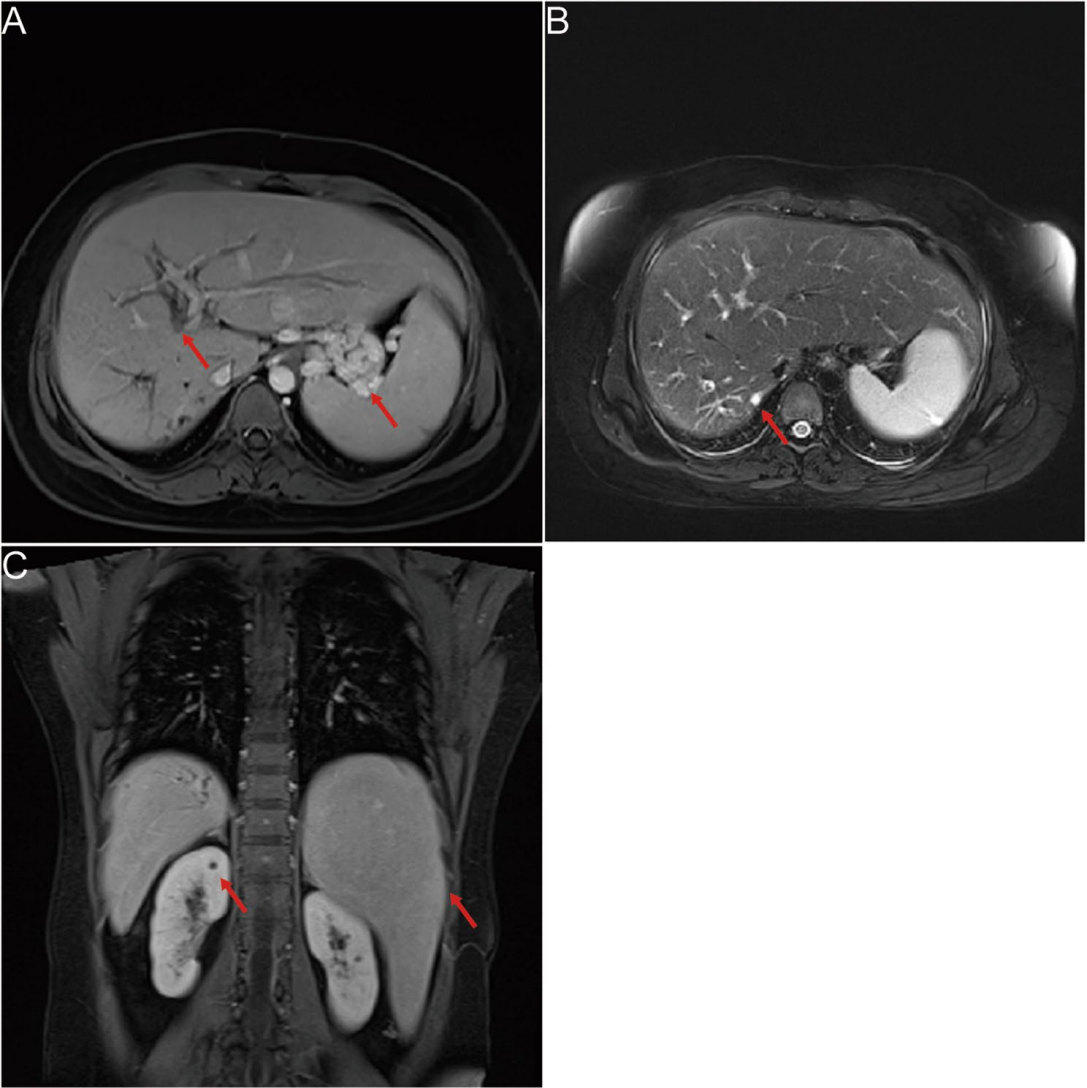
Imaging findings at discharge and follow-up results of the patient. **(A)** MRI imaging revealed intrahepatic bile duct dilatation and gastric fundic varices (arrow). **(B)** MRI imaging revealed cystic dilatation of the intrahepatic bile duct (arrow). **(C)** MRCP imaging showed the morphological characteristics of bile duct tree

Anti-bacterial therapy (Meropenem injection), anti-inflammatory therapy (Methylprednisolone Sodium Succinate injection), anti-diarrheal therapy (Montmorillonite powder and Loperamide Hydrochloride capsules), and platelet production-promoting therapy (Recombinant Human Thrombopoietin injection) were given. The patient was treated with for Loperamide for 1 day, anti- diarrheal therapy was stopped when symptom improved. After nearly one month of treatment, the levels of WBC, CRP, PCT, CA19-9/125, and liver fibrosis indexes returned to normal, and platelet count increased to 64 × 10^9/L. Biochemical tests revealed normal levels of ALT (16 U/L), AST (19 U/L), TBIL (12.4 µmol/L), DBIL (2.6 µmol/L), TBA (3.4 µmol/L), AKP (40 U/L), and SCr (72 µmol/L). Images revealed dilated intrahepatic bile ducts, full left lobe of liver, full gallbladder, enlarged spleen, and multiple varices in the esophagogastric fundus (Fig. 2 A-C).

**Fig. 3:**
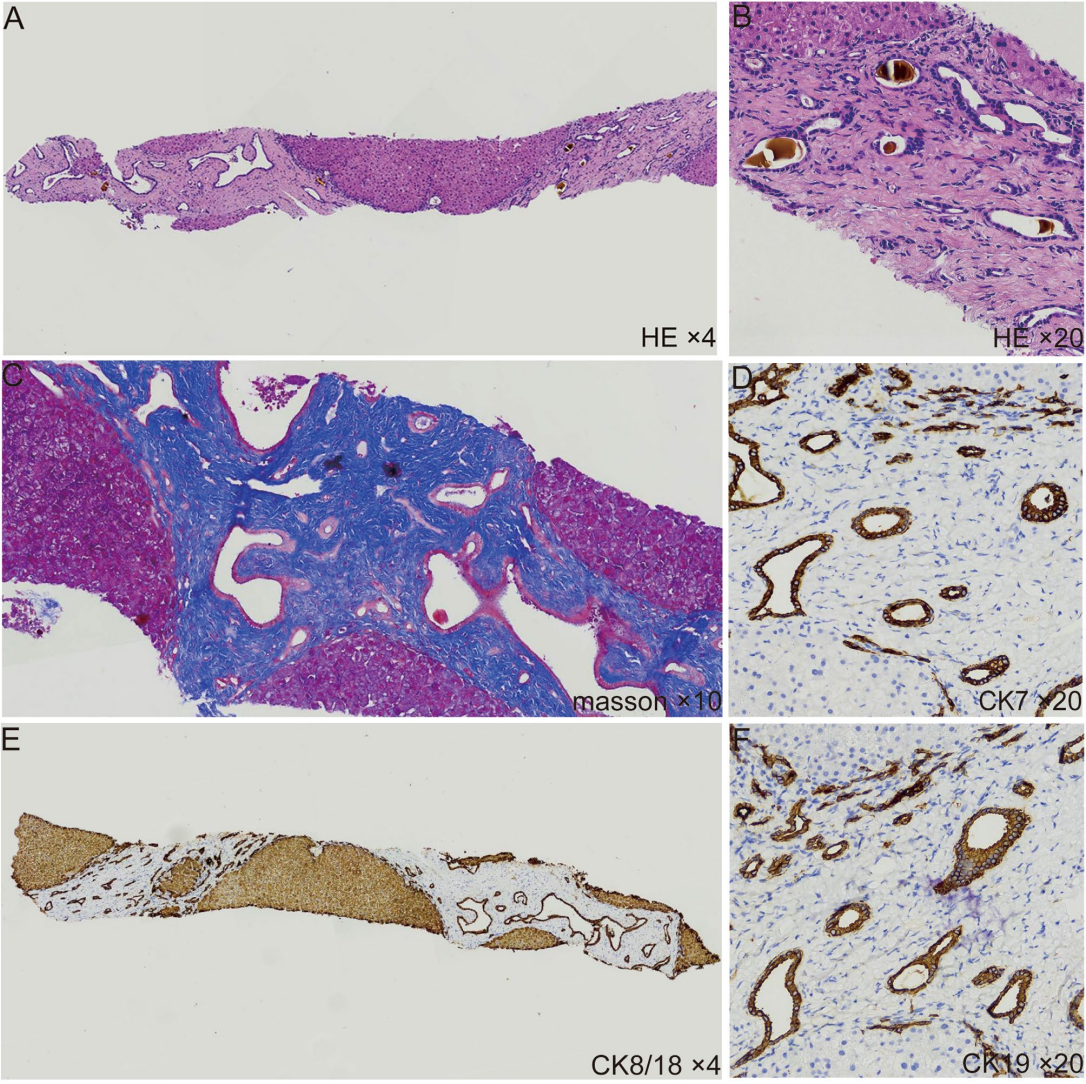
Liver biopsy of the patient. **(A-B)** H&E staining showed several portal tracts are interconnected by bridging fibrous septa, which contained intensive proliferation of the bile ducts, some of the bile ducts contained bile plugs **(C)** Masson staining revealed extensive formation of collagen fibers. **(D-F)** Immunohistochemistry CK7, CK8/18, and CK19 staining revealed a large amount of irregular bile duct, and bile duct cystic dilatation

The patient underwent liver biopsies after receiving anti-infective therapy. The pathological findings of the liver biopsy showed several portal tracts are interconnected by bridging fibrous septa, which contained intensive proliferation of the bile ducts without inflammatory cell infiltrates, some of the bile ducts contained bile plugs (Fig. 3 A-C). Immunohistochemical CK7 (Cytokeratin 7), CK8/18 (Cytokeratin 8/18), and CK19 (Cytokeratin 19) staining in the liver tissue showed massive hyperplasia of irregular bile ducts and cystic dilatation of bile ducts (Fig. 2 D-F).

**Fig. 4:**
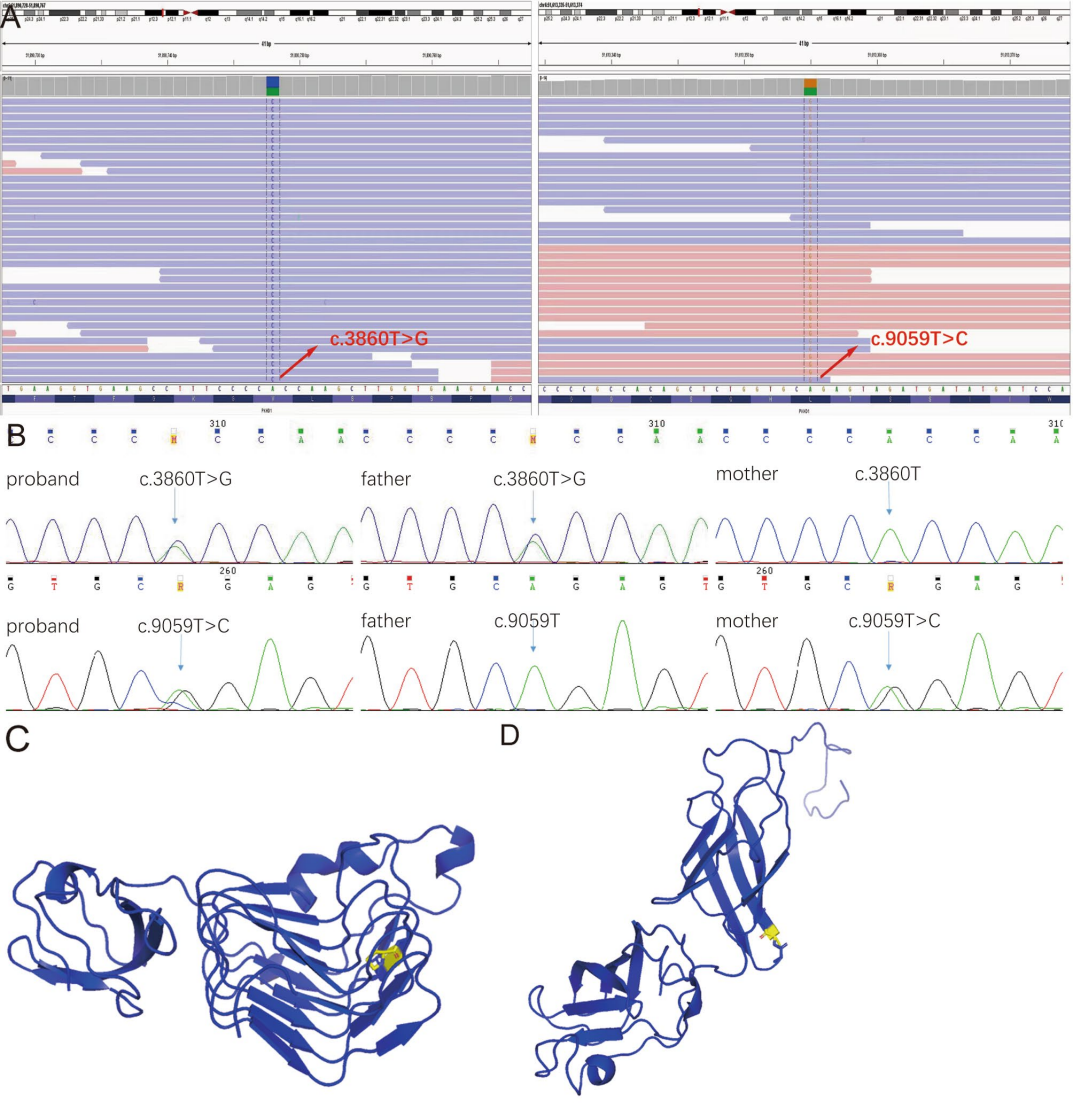
Analysis of gene mutation. **(A)** Whole-exome sequencing identified two heterozygous missense variants in the patient. **(B)** Sanger sequencing of the two targeted PKHD1 variations in the patient and her parents. The arrows indicate two heterozygous missense variants c.3860T > G and c.9059T > C. The arrows indicate her father carried c.3860T > G variant and were the wild-type genotype of c.9059T > C variant, her mother carried c.9059T > C variant and were the wild-type genotype of c.3860T > G variant. **(C-D)** Using PyMol to predicted the structure of PKHD1 with the variant PKHD1 of V1287G and p. L3020P, respectively. The difference in the side chains were indicated in yellow

Furthermore, whole-exome sequencing was performed, and compound heterozygous variants were identified in the PKHD1 gene (reference sequence NM_138694): two missense substitutions c.3860T > G (p. V1287G) and c.9059T > C (p. L3020P) (Fig. 4A). Both of the targeted variations were confirmed by Sanger sequencing in the index patient and her parents, c.3860T > G variant was paternally inherited, and c.9059T > C variant was maternally inherited (Fig. 4B). The in-silico analysis predicted the c. 3860T > G/p. V1287G variant as unknown clinical significance (SIFT score 0.029, damaging; Polyphen2 score 0.001, benign; REVEL score 0.226, tolerable; ClinPred score 0.02426, benign), and the c. 9059T > C/p. L3020P variant as deleterious (SIFT score 0.012, damaging; Polyphen2 score 0.984, probably damaging; REVEL score 0.841, damaging; ClinPred score 0.7198, pathogenic). Swiss Model homology modeling was performed to visualize three-dimensional structures with the two variations in the PKHD1 protein (Fig. 3C-D).

A diagnosis of CHF complicated with biliary sepsis was made based on the patient’s laboratory tests, imaging findings, pathological and genetic results. No morphological changes in the liver were observed comparing liver imaging during and after biliary sepsis. After being discharged from the hospital, she took a small dose of carvedilol (10 mg once daily), a vasodilating beta-blocker, used to reduce the portal venous pressure. After nearly three months of portal hypotensive treatment, she had no symptoms such as fever, abdominal pain, vomiting of blood, black stools, and bloody stools. The levels of inflammatory markers were normal, serum detections of liver and kidney function were normal, while platelet counts declined to 44 × 10^9/L.

## Discussion

Acute biliary conditions, including cholangitis, obstruction, and biliary leakage, may lead to severe biliary sepsis [6]. Apart from common diseases such as parasites, cholelithiasis, and bile duct malignancies, various congenital abnormalities of the biliary (including CHF and Caroli disease) can also lead to bile stasis and cholangitis. Due to the direct connection between the “biliary cyst” and the biliary tree, biliary stasis may promote the development of infection [7]. Acute cholangitis is a potentially fatal complication due to the risk of biliary sepsis. To date, a total of 9 CHF patients who developed sepsis have been reported, and 6 patients among these died [8–12] (Table 1). We present a young-female CHF case with biliary sepsis due to KP infection, after timely treatment, she is alive and her liver function returns to the normal.

**Table 1:** Clinical features and outcomes of CHF patients with sepsis reported

	Gender	Age	Liver	Sepsis	Bacteria	Outcome	Reference
1	Female	27 years	CHF	Yes	NA	Died	Tamura et al. [8]
2	Female	2 years	CHF + Caroli’s disease	Yes	NA	Died	Nakanuma et al. [10]
3	Female	20 years	CHF + Caroli’s disease	Yes	NA	Died	Nakanuma et al. [10]
4	Male	13 years	CHF	Yes	NA	Died	De Vos et al. [9]
5	Male	27 years	CHF	Yes	E. coli	Alive	De Vos et al. [9]
6	Male	7 years	CHF	Yes	E. coli	Alive	De Vos et al. [9]
7	Male	21 years	CHF	Yes	E. coli	Died	De Vos et al. [9]
8	Male	4 months	CHF	Yes	NA	Died	Bharani et al. [11]
9	Female	5 years	CHF	Yes	Klebsiella oxytoca	Alive	Alvarez et al. [12]

Our patient had a high level of CA19-9. CA19-9 is often used for bile duct cancer screening, and elevated CA19-9 level can also be seen in the setting of some biliary inflammatory diseases and benign biliary obstructive diseases [13]. The patient’s PET/CT imaging showed no evidence of malignant tumor. Her blood culture was positive for KP with increased levels of CRP and PCT. Therefore, the patient had biliary sepsis caused by KP infection. The level of the CA19-9 returned to normal promptly after anti-infection therapy. Meanwhile, the patient presented with portal hypertension (including splenomegaly and esophagogastric fundus varices), and kidney cysts. The patient’s imaging showed no irregularities or nodule liver surface with rounded liver margins, suggesting non-cirrhotic portal hypertension rather than true cirrhosis. Her liver biopsy strongly suggested that the patient had congenital hepatic fibrosis, characterized by periportal fibrosis with irregularly shaped proliferating bile ducts. Defects in ductal plate remodeling will lead to the formation of portal fibrotic tissue, and this portal fibrosis could result in the development of cholangitis or portal hypertension [14]. Assessing serum fibrosis indicators during an acute episode of cholangitis is not recommended as results will be affected by the presence of infection. And we also performed imaging examination and liver biopsy to confirm the patient’s liver fibrosis. Altogether, the diagnosis of CHF with biliary sepsis in our patient was made. Additionally, portal hypertension would promote bacterial translocation and reduce bacterial clearance by inducing the formation of shunts in the portal venous system [15]. Poor lifestyle, such as staying up late, and high oil and salt diet, may be a trigger for infection. Unhealthy Diet may lead to increased inflammation, and reduced control of infection [16]. Healthy Diet and Lifestyle Improve the Gut Microbiota and Help Combat Fungal Infection [17]. Obesity and lifestyle (including smoking and inactivity) are associated with an increased risk of bacterial infections [18, 19]. Our patient had a BMI 28 kg/m^2^ and had unhealthy habits, which may make her predispose to infections. However, we do not see dramatic progression of liver disease after anti-infective treatment in our patient. This clinical outcome in our patient could be attributed to the timely treatment and young age, as well as the pathogenicity of the genetic variants.

Genetic testing results showed that our patient carried compound heterozygous variants in *PKHD1*, paternally inherited c.3860T > G (p. V1287G) and maternally inherited c.9059T > C (p. L3020P). In silico analysis using SIFT, PolyPhen-2, REVEL, and ClinPred predict c.3860T > G variant to be damaging, benign, tolerable, and benign, respectively; and c.9059T > C variant to be damaging, probably damaging, damaging, and pathogenic, respectively. It is reported that patients with two truncating variants in *PKHD1* had perinatal death, and at least one missense variant was required for survival beyond the neonatal period [20]. Thus, disease progression is closely correlated with *PKHD1* variants. These two missense variants had been reported in previous papers [21, 22], providing evidences for the pathogenicity of the two variants. However, the above in silico analysis of these two missense variants predict to be of unknown significance and damage, respectively, suggesting a week pathogenicity. We suspected that the two variations carried by our patient may lead her in an early stage of CHF in her age. Consistently, her gastroscopy revealed fundic varices but without red signs.

There is currently no specific therapy that can stop or reverse the pathological process in CHF, the only curative treatment is hepatic transplantation [23]. The most difficult clinical challenge for CHF patients is managing portal hypertension and cholangitis. Pharmacological therapies, non-selective beta blockers are used in cirrhosis to reduce portal pressure, and ursodeoxycholic acid is used to treat severe cholestasis [24]. Thrombocytopenia (39 × 10^9/L) in our patient was due to a combination of sepsis and splenic dysfunction. Recombinant human thrombopoietin is effective in raising platelet counts in septic thrombocytopenic patients [25]. After recombinant human thrombopoietin was used, the platelet level in the patient was increased to 64 × 10^9/L. In this case, her gastroscopy revealed fundic varices without red signs, whereas the risk of gastrointestinal bleeding remains high. During the subsequent follow-up, she took carvedilol on a regular basis and had no gastrointestinal bleeding, and recurrent cholangitis. We suggested that the patient develop healthy life habits to avoid infections causing recurrence of cholangitis, and undergo routine blood tests, liver function tests and hepatobiliary imaging.

## Data Availability

The data have been deposited in the NCBI SAR repository, accession number SRR25064178, and will be released as soon as the paper is accepted.
